# Supplementary material for: Physical determinants of asymmetric cell divisions in the early development of Caenorhabditis elegans
Source: Sci Rep. 2017 Aug 24;7:9369. doi: 10.1038/s41598-017-09690-4 (PMC5571195; doi:10.1038/s41598-017-09690-4)

Physical determinants of asymmetric cell divisions in the early development of *Caenorhabditis elegans*

Rolf Fickentscher and Matthias Weiss

Experimental Physics I, University of Bayreuth, D-95440 Bayreuth, Germany

Supplementary Figure S1

(referenced and explained in Materials and Methods of the main article).

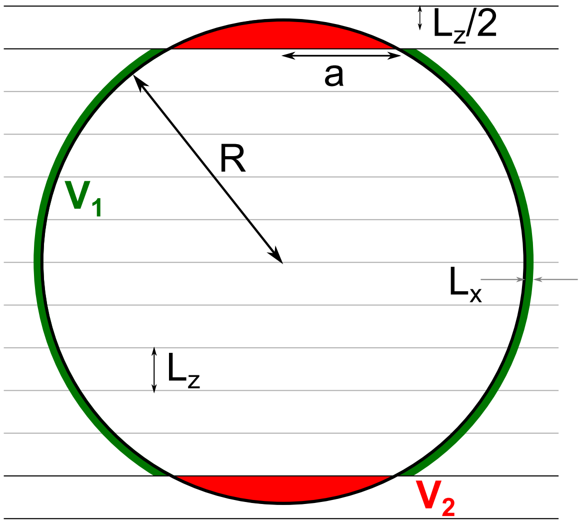

Supplementary Figure S2

Comparison of volumetric asymmetries in wild-type and eggshell-free embryos as shown in Fig. 2C but with error bars. We deliberately have used standard deviations for the error bars (not the smaller standard errors) to reflect the ensemble's fluctuations. For better readability, mean values are shown as horizontal lines instead of the bar charts used in Fig. 2C.

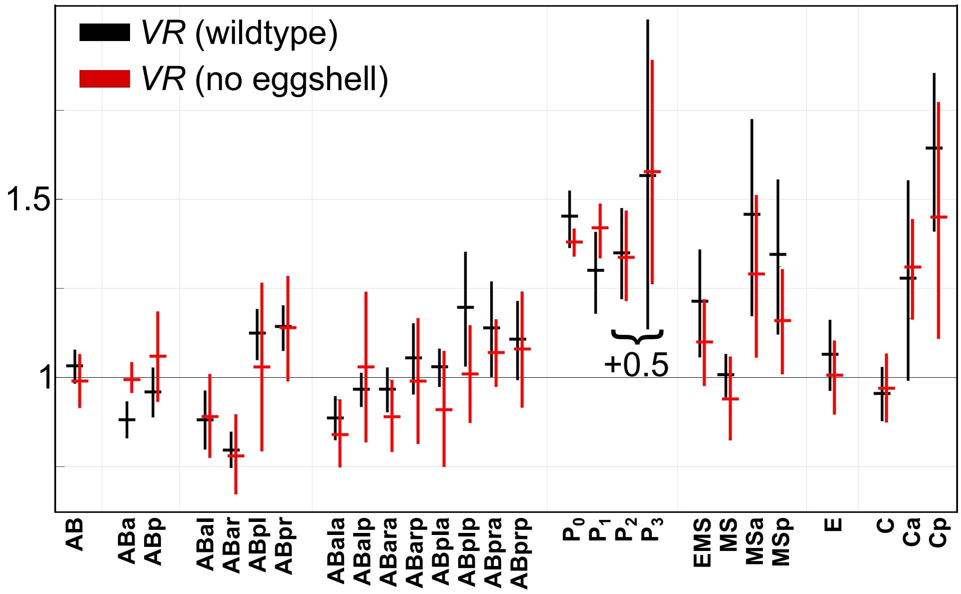

Supplement: Supplementary file 1 — Supplementary information [file 41598_2017_9690_MOESM1_ESM.pdf]
